# Stakeholder attitudes to the regulation of traditional and complementary medicine professions: a systematic review

**DOI:** 10.1186/s12960-021-00579-y

**Published:** 2021-03-29

**Authors:** Jenny Carè, Amie Steel, Jon Wardle

**Affiliations:** 1 Australian Research Centre in Complementary and Integrative Medicine, University of Technology Sydney, Ultimo, NSW 2007 Australia; 2 National Centre for Naturopathic Medicine, Southern Cross University, Lismore, NSW 2480 Australia

**Keywords:** Complementary medicine, Complementary and alternative medicine, Credentialling, Licensure, Metasynthesis, Professionalisation, Regulation, Registration, Systematic review, Traditional and complementary medicine

## Abstract

**Background:**

There has been a considerable increase in the number of traditional and complementary medicine (T&CM) practitioners over the past 20 years and in some jurisdictions are estimated to outnumber general practitioners. Despite this globally significant role, it is apparent that worldwide not all T&CM professions operate under adequate accountability and regulatory oversight for maintaining public protection. To date there has been no published systematic examination of stakeholder opinions regarding regulated and unregulated T&CM occupations. In response, this review aims to investigate, describe, and analyse attitudes held by a range of stakeholder groups towards the regulation of T&CM professions.

**Methods:**

A database search of AMED, CINAHL, Embase, Ovid MEDLINE, ProQuest, PsycINFO, PubMed, Scopus, and Google Scholar was conducted for original research published between 2000 and 2020 on stakeholder opinions regarding the regulation of T&CM professions.

**Results:**

Sixty studies across 15 countries reported on the views of six health care stakeholder groups: consumers, T&CM practitioners, conventional medicine practitioners, professional associations, education providers, and policy-makers. Across all stakeholder groups there was between 15% and 95% (median 61%) support for, and 1% to 57% (median 14%) opposition to the regulation of various T&CM professions. The main reasons for supporting regulation included providing greater public protection, raising training and practice standards, establishing title protection, and gaining acceptance from conventional medicine providers. Concerns regarding regulation included potential restrictions to practice, misappropriation of practice, and medical oversight of T&CM practitioners. Few studies canvassed the views of professional associations (*n* = 6), education providers (*n* = 2), and policy-makers (*n* = 2).

**Conclusions:**

There appears to be broad support for the regulation of T&CM professions, although there was wide variation in attitudes as to how this should be applied. Further research, with a particular focus on policy-makers, education providers, and professional associations, is critical to inform appropriate health policy and practice recommendations relating to T&CM professional regulation across jurisdictions. Systematic review registration: the a priori protocol for this systematic review was registered in PROSPERO and is available at: www.crd.york.ac.uk/PROSPERO/display_record.asp?ID=CRD42020198767.

## Background

Health care systems are coming under increasing pressure from challenges posed by the growing burden of non-communicable diseases [1, 2], the health care needs of ageing populations [3], the accelerating incidence of epidemics and pandemics [4], burgeoning health care costs [5], and the prospect of health care workforce shortages [6]. Changing needs, shifting priorities and the increasingly consumer-led nature of health care systems have resulted in significant changes in the contemporary health care workforce. This includes the evolution or growth of new professions and changes in scope of existing professions. According to the World Health Organization (WHO) there are now over 150 occupations in the health workforce sector [7], although one commentator considers this number closer to 350 [8].

One area of the health workforce in which there has been considerable growth and evolution is traditional and complementary medicine (T&CM). T&CM refers to a broad set of health care practices and beliefs indigenous to a culture and place (traditional medicine), and practices that are neither indigenous nor part of the predominant health system of a country (complementary) [9, 10]. T&CM designations are therefore jurisdictionally defined [9]. Although some T&CM practices have ancient roots, the worldwide growth in consumer use and recognition of T&CM commenced in the latter part of the twentieth century, in part due to the Declaration of Alma-Ata [11] and later the release of WHO traditional medicine strategies [9, 12]. The rise in T&CM use has proved to be a global phenomenon, evident in both developing and developed nations [13–20]. Commensurate with this rise in use there has been a substantial increase in the number of T&CM practitioners over the past 20 years [21–23]. Indeed in some jurisdictions the T&CM cadre is estimated to outnumber general practitioners (GPs) [23], making this cohort a significant part of the health care workforce globally.

Regulation of health practitioners is generally defined as the actions taken by public authorities to control activities and standards relating to health practice [24–26]. While models of health workforce regulation vary across jurisdictions [27, 28], one schema classifies six categories of occupational licensing: no regulation, self-regulation, state sanctioned self-regulation, statutory self-regulation, co-regulation and statutory regulation [29]. In many jurisdictions the regulation of health professions appears to be moving away from non-government regulatory models towards nationally based regulatory approaches [30], and greater regulatory partnerships between the public, professions, and regulators [27]. Further, the WHO has identified regulation as a key milestone of their global health workforce strategy [6], and regulation of the T&CM workforce specifically as one of three strategic objectives of the WHO T&CM strategy [9]. Despite these strategic priorities, there is considerable variation worldwide in the way in which T&CM occupations are regulated, as well as the form of regulation applied. Regulatory developments for T&CM practices are argued to be lagging behind their growth in use [31], and not all T&CM occupations operate within adequate accountability-based, public interest regulatory frameworks [9, 10]. For instance, some T&CM professions are statutorily regulated in certain countries, others reside outside statutory frameworks but occupy a state-recognised place in health provision, and some T&CM practices are neither statutorily regulated nor acknowledged by the state, but continue to operate within their jurisdictions, sometimes informally [10].

There is a public health imperative for governments to establish mechanisms for recognising and monitoring T&CM practices and practitioners, and promote their appropriate integration or restriction within health care systems [10]. Establishing suitable regulatory frameworks may ensure appropriate and consistent minimum standards of education and practice [10, 32], and facilitate workforce mobility across country borders [9], potentially alleviating forecast health care workforce shortages [6], and contributing to the WHO’s mission of promoting health for all [33]. WHO, through its traditional medicine strategy, has noted the lack of action in progressing T&CM regulation and encourages member states to engage more actively with this policy to facilitate the appropriate regulation of T&CM within their jurisdictions [9]. By taking a global perspective the development of insights regarding potential enablers and barriers of regulation across a range of jurisdictions is facilitated, which can inform future application of regulatory policy in a number of different settings.

Despite widespread consumer utilisation of T&CM, the broadening reach of these practices, and the increasing tendency to regulate T&CM professions, what remains unknown and requires greater understanding are the attitudes and perceptions towards regulation of T&CM across the health care stakeholder landscape. In the broadest sense, stakeholder attitudes are important considerations in many contexts and settings [34]. Within the health care context, understanding stakeholder attitudes is important to ensure that regulation is sustainable, responsive, and appropriate, and serves the public interest in a manner that is reflective of societal norms, expectations, and practices. Attitudes are shaped by self-interest, social identification, and personal values through which opinions are formed [35]. Attitudes and opinions have a bearing on policy by influencing regulatory and policy agendas [35–37]. Disregarding the attitudes of key stakeholders risks privileging the views of certain groups at the expense of others [36, 37] and may result in regulatory developments that are not responsive to changing health workforce requirements. To date there has been no systematic examination of stakeholder opinions regarding regulated and unregulated T&CM occupations, a deficiency this systematic review aims to address. Consistent with regulatory trends, this review takes an expansive view across a range of stakeholder groups and jurisdictions to investigate, describe, and analyse attitudes towards the regulation of T&CM professions.

## Methods

### Review protocol

In order to inform the development of evidence-based policy, the objective of this review was to investigate, describe, and analyse all available stakeholder attitudes regarding T&CM regulation canvassed over the past 20 years, classifying and reporting the data according to emergent stakeholder groupings.

The review protocol was developed in accordance with ‘Assessing the Methodological Quality of Systematic Reviews’ (AMSTAR) guidelines [38] and the ‘Preferred Reporting Items for Systematic Review and Meta-Analysis Protocols’ (PRISMA-P) 2015 checklist [39]. It was registered in PROSPERO (#CRD42020198767) [40] prior to completing the literature search.

### Search strategy

Searches were conducted in eight databases (AMED, CINAHL, Embase, Ovid MEDLINE, ProQuest, PsycINFO, PubMed, Scopus) between 22/05/2020 and 26/05/2020, supplemented by a Google Scholar search 26/06/2020 to 28/06/2020. The search strategy consisted of free text and medical subject heading search terms. T&CM terms were developed using the Cochrane Complementary Medicine Glossary of CAM terms [41] as well as a selection of commonly used terms within T&CM professions and practices [42]. Regulation-related terms were developed by the first author (JC) from background reading of research regarding health care regulation [31, 43–45]. Search terms were modified to suit the Google Scholar interface. The research team has published multiple systematic literature reviews related to health policy and T&CM, and a librarian was consulted in the development of the database search protocols. Table 1 provides the search terms used for Ovid MEDLINE. The full search protocol is available at: https://www.crd.york.ac.uk/PROSPEROFILES/198767_STRATEGY_20200714.pdf.

**Table 1:** Search terms used in Ovid MEDLINE for attitudes to the regulation of traditional and complementary medicine professions

	Traditional and complementary medicine	Regulation
1	Exp complementary therapies/	(Accreditation adj20 (profession* OR practitioner)).mp
2	Complementary medicine.mp	(Certification adj20 (profession* OR practitioner)).mp
3	Complementary therap*.mp	(Consumer protection adj20 (profession* OR practitioner)).mp
4	Alternative medicine.mp	Credential?ing.mp
5	Alternative therap*.mp	Government regulation/
6	Natural medicine.mp	Government regulation.mp
7	Natural therap*.mp	(Healthcare reform adj20 (profession* OR practitioner)).mp
8	Acupuncture/	(Health care reform adj20 (profession* OR practitioner)).mp
9	Acupunctur*.mp	Health care regulation.mp
10	Aromatherapy/	Healthcare regulation.mp
11	Aromatherap*.mp	(Health policy adj20 (profession* OR practitioner)).mp
12	Ayurved*.mp	Legislation/
13	Chiropractic/	(Legislati* adj20 (profession* OR practitioner)).mp
14	Manipulation chiropractic/	Licensure/
15	Chiropract*.mp	(Licensure adj20 (profession* OR practitioner)).mp
16	Herbal medicine/	Occupation* registration.mp
17	Herbalis*.mp	Occupation* regulation.mp
18	Herbal medicine practitioner*.mp	Professionali?ation.mp
19	Phytotherap*.mp	(Registration adj20 (practitioner OR profession*)).mp
20	Homeopath*.mp	(Regulation adj20 (practitioner OR profession*)).mp
21	Homoeopath*.mp	Risk governance.mp
22	Massage/	Risk perception.mp
23	Massage.mp	Risk understanding.mp
24	Naturopath*.mp	Statutory registration.mp
25	Osteopathic physicians/	Statutory regulation.mp
26	Osteopathic medicine/	
27	Osteopath*.mp	
28	Exp medicine, east asian traditional/	
29	Traditional chinese medicine.mp	
30	Traditional medicine.mp	
31	African healing.mp	
32	African medicine.mp	
33	Arabic healing.mp	
34	Arabic medicine.mp	
35	Indian healing.mp	
36	Indian medicine.mp	
37	Japanese medicine.mp	
38	Japanese healing.mp	
39	Kampo medicine.mp	
40	Korean medicine.mp	
41	Sidda medicine.mp	
42	Tibetan medicine.mp	
43	Unani medicine.mp	
44	Yunani medicine.mp	

### Selection criteria

Studies were included if they were original research, in English, published between 2000 and June 2020. A stakeholder attitude map [34] was used to conceptually consider categories of stakeholders within a health care context. Specific stakeholder groups were not defined a priori. All available stakeholder research canvassing views regarding T&CM practitioner regulation was accepted for inclusion. From background reading and consideration of stakeholder categories [34] the following groups were expected to feature in the search results: consumers, T&CM practitioners, conventional medicine practitioners, professional associations, education providers, and policy-makers. Defining T&CM professions applicable to all jurisdictions was problematic [46, 47], hence this review accepted the classification applied by each included study. Review articles, narrative research, commentaries, editorials, and non-English language studies were excluded.

### Study selection

Retrieved records were imported into EndNote X9 (Clarivate Analytics 2018) by JC. Records were deduplicated, titles and abstracts were screened, and resulting full texts were scrutinised by JC. Those meeting the selection criteria were accepted for inclusion. Reference lists of included manuscripts, and all referenced systematic reviews, were manually searched by JC for additional relevant titles. A proportion of records (10%) was reviewed at each screening stage by all members of the research team (AS, JC, JW). Any discrepancies regarding inclusion eligibility were resolved through discussion.

### Data extraction and appraisal

A data extraction table was developed in Microsoft Excel® (Microsoft 365) to capture the attributes of interest. The table was established by the research team based on the research aim and informed by previous systematic reviews. It was piloted through the collection of attributes of interest, was developed iteratively, and modified by the research team as data extraction proceeded. Data were recorded by JC from detailed reading of included studies during which the relevant data were transferred to the data table and subsequently verified by the research team.

Identified studies were appraised for risk of bias. Cross-sectional observational studies adopting qualitative research designs were assessed using the Joanna Briggs Institute Critical Appraisal Checklist for Qualitative Research [48]. Cross-sectional observational studies employing quantitative research methods were appraised using Hoy et al.’s checklist for population-based prevalence studies [49]. The assessments were conducted by JC, and a sample of studies was reviewed by the research team. As this was the first systematic review of this topic, the authors considered it appropriate to include the entirety of available research conducted over the past 20 years irrespective of assessment outcomes.

### Data synthesis and analysis

A meta-analysis was not possible due to significant heterogeneity between studies. Where quantitative data were available this is summarised and narratively analysed. Qualitative data were analysed, categorised inductively, and reported narratively based on themes that emerged from the data in the identified studies. Stakeholder categorisation was undertaken inductively.

Throughout this review, the term regulation refers to the statutory/legislative governance of health care occupations or the registration of practitioners, unless otherwise stated.

## Results

**Fig. 1:**
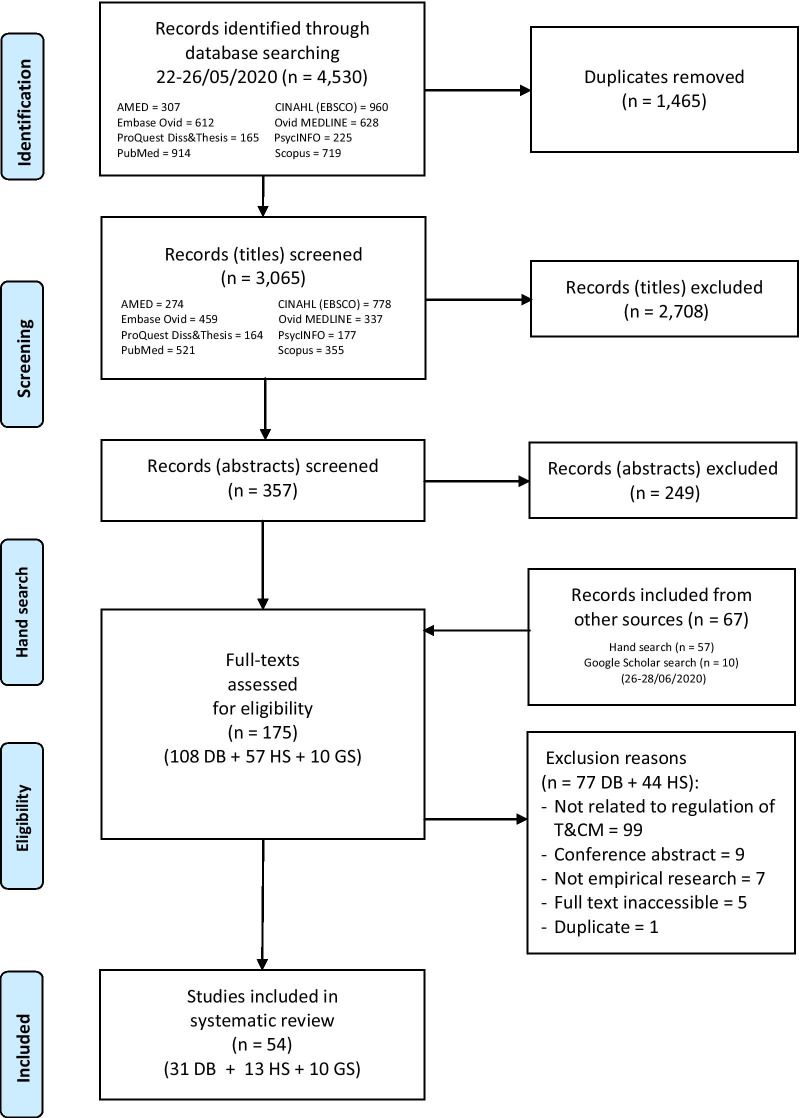
PRISMA-P flowchart of study selection. *DB* database search, *HS* hand search, *GS* Google Scholar search

The database, Google Scholar and manual searches yielded 3132 non-duplicated records. Following screening, a total of 54 published and unpublished papers met the inclusion criteria and were selected for review. The reasons for study exclusion are detailed in Fig. 1.

### Risk of bias

Two studies were excluded from critical appraisal because they were qualitative analyses of open-ended questions that were part of larger, and separate, quantitative studies [50, 51]. One report included three separate stakeholder studies [52] which were assessed individually. Therefore, a total of 54 studies were appraised.

#### Cross-sectional observational studies using qualitative research design methods (*n* = 21)

Six studies met the appraisal requirements for the first five domains [53–58]. A further six adequately addressed domain 6 [55–60]. Most studies (*n* = 16) addressed domain 7 [53–70], and all studies met the requirements of the final three domains, except for one which did not address research ethics [63]. The overall risk of bias was considered moderate in 17 studies [52–55, 59, 61–72] and low in the remaining four [56–58, 60].

See Table 2 for full details of critical appraisal of qualitative studies.

**Table 2:** Risk of bias assessment for qualitative studies

	Domains										
First author year	1	2	3	4	5	6	7	8	9	10	Overall summary (risk)
Barnes 2018 [61]	U	U	U	U	U	N	Y	Y	Y	Y	Include (moderate)
Boon 2004 [53]	Y	Y	Y	Y	Y	N	Y	Y	Y	Y	Include (moderate)
Canaway 2009 [71]	U	U	U	U	U	N	N	Y	Y	Y	Include (moderate)
Cavaco 2017 [72]	U	U	U	U	U	N	N	Y	Y	Y	Include (moderate)
Clarke 2004 [54]	Y	Y	Y	Y	Y	N	N	Y	Y	Y	Include (moderate)
Ericksen-Pereira 2020 [55]	Y	Y	Y	Y	Y	Y	N	Y	Y	y	Include (moderate)
Flower 2015 [62]	U	U	U	U	U	N	Y	Y	Y	Y	Include (moderate)
Gilmour 2002 [63]	U	U	U	U	U	N	Y	Y	N	Y	Include (moderate)
Gyasi 2017 [56]	Y	Y	Y	Y	Y	Y	Y	Y	Y	Y	Include (low)
James 2020 [59]	U	U	U	U	U	Y	Y	Y	Y	Y	Include (moderate)
Jarvis 2015 [64]	U	U	U	U	U	N	Y	Y	Y	Y	Include (moderate)
Kelly 2005 [65]	U	U	U	U	U	N	Y	Y	Y	Y	Include (moderate)
Kelner 2004 [66]	U	U	U	U	U	N	Y	Y	Y	Y	Include (moderate)
Kelner 2004 [67]	U	U	U	U	U	N	Y	Y	Y	Y	Include (moderate)
Kelner 2006 [68]	Y	U	U	U	U	N	Y	Y	Y	Y	Include (moderate)
Lin (Section 9, Hill) 2005 [52]	U	U	U	U	U	N	N	Y	Y	Y	Include (moderate)
Malhotra 2020 [57]	Y	Y	Y	Y	Y	Y	Y	Y	Y	Y	Include (low)
Smith 2015 [58]	Y	Y	Y	Y	Y	Y	Y	Y	Y	Y	Include (low)
Steel 2020 [60]	U	U	U	U	U	Y	Y	Y	Y	Y	Include (low)
Wardle 2013 [69]	U	U	U	U	U	N	Y	Y	Y	Y	Include (moderate)
Welsh 2004 [70]	U	U	U	U	U	N	Y	Y	Y	Y	Include (moderate)

**Domains**

1. Is there congruity between the stated philosophical perspective and the research methodology?

2. Is there congruity between the research methodology and the research question or objectives?

3. Is there congruity between the research methodology and the methods used to collect data?

4. Is there congruity between the research methodology and the representation and analysis of data?

5. Is there congruity between the research methodology and the interpretation of results?

6. Is there a statement locating the researcher culturally or theoretically?

7. Is the influence of the researcher on the research, and vice-versa, addressed?

8. Are participants, and their voices, adequately represented?

9. Is the research ethical according to current criteria or is there evidence of ethical approval by an appropriate body?

10. Do the conclusions drawn in the research report flow from the analysis, or interpretation, of the data?

N = criterion not adequately met; Y = criterion adequately met; U = unclear if criterion met

#### Cross-sectional observational studies using quantitative research design methods (*n* = 33)

Descriptive studies were at the greatest risk of bias in the first four domains where only nine adequately addressed all items [52, 73–80]. All studies met the requirements of domains 5 and 6. Studies performed reasonably well for domains 7 and 8. Domain 9 was considered irrelevant because all studies employed survey methods. Seventeen studies failed to address the final domain [74, 77, 79, 81–94]. Overall, three studies were rated as high risk [85, 91, 92], 16 were considered moderate risk [81, 82, 84, 86–90, 93–100], and 14 studies were judged as low risk [52, 73–80, 83, 101–104].

See Table 3 for full details of critical appraisal of quantitative studies.

**Table 3:** Risk of bias assessment for quantitative studies

	External validity domains				Internal validity domains						
First author year	1	2	3	4	5	6	7	8	9	10	Overall risk
Al Mansour 2015 [95]	N	N	N	Y	Y	Y	Y	Y	N/A	Y	Moderate
Bensoussan 2004 [73]	Y	Y	Y	Y	Y	Y	Y	Y	N/A	Y	Low
Braun 2013 [74]	Y	Y	Y	Y	Y	Y	Y	Y	N/A	N	Low
Chaterji 2007 [81]	N	N	N	N	Y	Y	Y	Y	N/A	N	Moderate
Cohen 2005 [101]	N	Y	Y	N	Y	Y	Y	Y	N/A	Y	Low
Cottingham 2015 [96]	N	N	N	Y	Y	Y	Y	Y	N/A	Y	Moderate
Cottingham 2017 [97]	N	N	N	Y	Y	Y	Y	Y	N/A	Y	Moderate
Cottingham 2018 [82]	N	N	N	Y	Y	Y	Y	Y	N/A	N	Moderate
Dooley 2010 [75]	Y	Y	Y	Y	Y	Y	Y	Y	N/A	Y	Low
El-Olemy 2014 [102]	N	Y	Y	Y	Y	Y	Y	Y	N/A	Y	Low
Emslie 2002 [76]	Y	Y	Y	Y	Y	Y	Y	Y	N/A	Y	Low
Evans 2008 [83]	Y	Y	Y	N	Y	Y	N	Y	N/A	N	Low
Flatt 2013 [77]	Y	Y	Y	Y	Y	Y	Y	Y	N/A	N	Low
Hall 2000 [103]	Y	Y	N	N	Y	Y	Y	Y	N/A	Y	Low
Harris 2006 [84]	Y	Y	Y	N	Y	Y	N	Y	N/A	N	Moderate
Langworthy 2000 [85]	N	N	N	N	Y	Y	Y	Y	N/A	N	High
Lin (Section 5, McCabe) 2005 [52]	Y	Y	Y	Y	Y	Y	Y	Y	N/A	Y	Low
Lin (Section 6, McCabe) 2005 [52]	Y	Y	Y	Y	Y	Y	Y	Y	N/A	Y	Low
Livingston 2010 [86]	Y	Y	Y	N	Y	Y	Y	Y	N/A	N	Moderate
Montbriand 2000 [87]	N	N	Y	N	Y	Y	Y	Y	N/A	N	Moderate
Morin 2017 [88]	Y	Y	Y	N	Y	Y	Y	Y	N/A	N	Moderate
Parker 2013 [78]	Y	Y	Y	Y	Y	Y	Y	Y	N/A	Y	Low
Poreddi 2016 [104]	Y	N	Y	Y	Y	Y	Y	Y	N/A	Y	Low
Poynton 2006 [98]	Y	Y	N	N	Y	Y	Y	Y	N/A	Y	Moderate
Price 2004 [89]	Y	Y	Y	N	Y	Y	N	Y	N/A	N	Moderate
Semple 2006 [90]	Y	Y	Y	N	Y	Y	Y	Y	N/A	N	Moderate
Taylor 2003 [91]	N	N	N	N	Y	Y	N	Y	N/A	N	High
Tiralongo 2010 [99]	Y	Y	Y	N	Y	Y	Y	N	N/A	Y	Moderate
Tsai 2008 [92]	N	N	N	N	Y	Y	Y	Y	N/A	N	High
Xue 2005 [93]	Y	Y	Y	N	Y	Y	N	Y	N/A	N	Moderate
Yu 2015 [100]	N	Y	N	N	Y	Y	Y	N	N/A	Y	Moderate
Zhang 2006 [94]	Y	Y	N	Y	Y	Y	Y	Y	N/A	N	Moderate
Zhang 2008 [79]	Y	Y	Y	Y	Y	Y	Y	Y	N/A	N	Low

**Domains**

1. Was the study’s target population a close representation of the national population in relation to relevant variables?

2. Was the sampling frame a true or close representation of the target population?

3. Was some form of random selection used to select the sample, OR was a census undertaken?

4. Was the likelihood of nonresponse bias minimal?

5. Were data collected directly from the subjects (as opposed to a proxy)?

6. Was an acceptable case definition used in the study?

7. Was the study instrument that measured the parameter of interest shown to have validity and reliability?

8. Was the same mode of data collection used for all subjects?

9. Was the length of the shortest prevalence period for the parameter of interest appropriate?

10. Were the numerator(s) and denominator(s) for the parameter of interest appropriate?

N = criterion not adequately met; Y = criterion adequately met; N/A = criterion not applicable

### Study characteristics

The 54 included studies consisted of one book chapter [53], two government/industry sponsored reports [52, 75], three doctoral/master’s theses [58, 78, 94], and 48 journal articles [50, 51, 54–57, 59–77, 79, 81–93, 95–104]. Four papers examined two stakeholder groups and were included separately in this review [70, 88, 91, 100]. One report [52], which was published in summarised form [105], consisted of five separate stakeholder studies, two of which were published in their entirety in scholarly journals [73, 101]. The published version of a third study [80] did not include the full data set relating to regulation. This study and the two remaining studies have been included in this review using the data from the unpublished report [52]. Contact with the corresponding authors of the book chapter, government/industry reports, and theses confirmed their research has not been published in any journal. Overall, the papers selected for inclusion were 60 separate studies from 54 original publications.

Twenty-four studies employed qualitative design methods, and 36 used quantitative methods. The studies spanned 15 countries: Australia (*n* = 19), Canada (*n* = 13), New Zealand (*n* = 10), UK (*n* = 5), Korea (*n* = 2), USA (*n* = 2), and Egypt, Ghana, India, Netherlands, Portugal, Saudi Arabia, Sierra Leone, South Africa, and Taiwan (*n* = 1 each). Six stakeholder groups were investigated: consumers (*n* = 8), T&CM practitioners (*n* = 19), conventional medicine practitioners (n = 23), professional associations (n = 6), education providers (n = 2), and policy-makers (*n* = 2). The T&CM occupations under study encompassed 36 discrete professions, the most common being naturopathy (*n* = 26), homeopathy (*n* = 22), acupuncture (*n* = 21), herbal medicine (*n* = 21), chiropractic (*n* = 18), and traditional Chinese medicine (*n* = 13). Nine studies did not specify the occupation, using terms such as alternative therapies, complementary and alternative medicine, traditional healing, as well as traditional and complementary medicine.

Table 4 provides details of study characteristics and findings.

**Table 4:** Study characteristics and attitudes regarding regulation of traditional and complementary medicine professions

First author year	Country (jurisdiction, if limited)	Study design	Stakeholder population	Sample *N*	T&CM profession/s examined	Main findings
**Consumers (** ***n* = 8)**						
El-Olemy 2014 [102]	Egypt (Tanta, Gharbiya governorate)	Quantitative Cross-sectional self-administered, supervised questionnaire survey	Students (University School of Education)	187	Traditional and complementary medicine	95.2% agreed that regulating practices was essential, 1.6% disagreed, 3.2% were uncertain Respondents with previous knowledge of T&CM (*n* = 146) were significantly more positive towards T&CM regulation (*p* = 0.002) 95.2% agreed that T&CM practices should be available and easily accessible, 92.0% agreed that integration of T&CM practices into health care improves patient care
Emslie 2002 [76]	Scotland (Grampian)	Quantitative. Population-based postal questionnaire survey	Residents	424	Acupuncture, aromatherapy, chiropractic, herbalism, homeopathy, hypnotherapy, osteopathy, reflexology	90% agreed that a register of approved therapists was essential/desirable (61% essential, 29% desirable), 1% agreed it was unnecessary, 8% gave no opinion 44% of 82 participants indicated concern about the registration of therapists 65% indicated concern about practitioner qualifications
Evans 2008 [83]	New Zealand (Gisborne)	Quantitative. Population-based face to face questionnaire survey	Inpatients of a provincial hospital	92	Acupuncture, antioxidants, aromatherapy, Bach flower remedies, Bowen, chiropractic, colour therapy, detoxification programs, dietary therapy, electro/biomagnetic therapy, herbal therapies, homeopathy, hypnotherapy, imagery/visualisation, iridology, massage (Romi Romi), naturopathy, osteopathy, reflexology, relaxation techniques, Rongoā Māori, shark cartilage, spiritual healing, vitamins, yoga	78% agreed T&CM should be regulated, e.g. like pharmaceutical drugs, a consultation with a qualified person first before purchasing medicines Reasons for supporting regulation were that medicines could be dangerous, treatment may not be safe, regulation may give consumers more access to information about products, and give better informed choice Reasons for opposing regulation were loss of freedom of choice, losing control over one's own health, and would probably make treatment too expensive
Lin 2005 (Section 9, Hill) [52] (Summarised in [105])	Australia (Melbourne)	Qualitative Focus groups	Consumers within a metropolitan area	24	Western herbal medicine, naturopathy	The majority of participants indicted there should be some form of regulation Because consumers place a great deal of trust in practitioners, and are often vulnerable, several participants indicated practitioners should have a qualification (implying an approved qualification) and that they should be regulated Those agreeing with regulation said it was needed to raise the standard of practitioners, ensure consistency of care, and stop unethical practice Regulation implied recognition of practices Some were aware that regulation did not ensure quality care, but thought that it was important for consumers to know that a practitioner had undertaken a minimum standard of training Concerns included the ability of professional associations to investigate complaints against their members, the possibility that poor practitioners could leave (or be forced to leave) one association only to join another, and the need for a body to hear complaints A few though regulation would not improve practitioner quality, and may inhibit them from trying new treatments, that an intuitive approach might be lost if practitioners were required to be registered, and that registration might restrict what they could practise. It was believed that regulation would not address the critical issue of practitioner communication skills
Taylor 2003 [91]	New Zealand (Wanganui)	Quantitative Print-based questionnaire survey	Consecutive patients of general practitioners at three general practices	104	Acupuncture, aromatherapy, chiropractic, hypnosis, Rongoā Māori	71.1% agreed regulation of T&CM practices should be on a par with orthodox medicine Patients indicated T&CM was sometimes, usually, or always safe 64.4% agreed there could be side-effects, and the majority were aware that there could be interactions with orthodox medicine
Xue 2005 [93]	Australia (Melbourne)	Quantitative Print-based questionnaire survey. Convenience sampling	Members of the public in three localities, predominantly Asian and Caucasian	575	Chinese medicine	76.3% agreed the practice should be regulated the same as Western medicine 67.7% agreed the practice should be more rigorously regulated 20.9% agreed registration improved public confidence in the practice as a health care option 18.7% agreed registration protected the public from unqualified service 37.9% agreed they would contact the registration board about service provision concerns 78.2% were aware statutory practitioner registration was recently introduced 29.9% agreed they only see a registered practitioner 80.8% agreed they would see a non-registered practitioner
Zhang 2006 [94]	Australia	Quantitative. Population-based random digit dialled telephone survey	Representative proportions of households in all states and territories	1067	Chinese medicine (Chinese herbal medicine, acupuncture)	86.4% agreed with the government regulation of acupuncture practitioners 86.0% agreed that mandatory registration of acupuncture practitioners provided for greater public safety and confidence in acupuncture 85.0% agreed with the government regulation of Chinese herbal medicine practitioners 82.7% agreed that mandatory registration of Chinese herbal medicine practitioners provided for greater public safety in Chinese herbal medicine
Zhang 2008 [79]	Australia (Victoria)	Quantitative Cross-sectional population-based random digit dialled telephone survey	Households in eight geographical regions	2526	Indian herbal medicine (Ayurveda), traditional Chinese herbal medicine, naturopathy, Western herbal medicine	89.6% agreed practitioners should be statutorily regulated (as for medical practitioners), 4.8% disagreed, 5.5% were unsure/did not answer 46.6% of herbal medicine users agreed they were aware of the potential risks of herbal medicine
**Traditional and complementary medicine practitioners (*n* = 19)**						
Bensoussan 2004 [73]	Australia	Quantitative. National postal questionnaire survey	Naturopaths, Western herbal medicine practitioners, homeopaths, nutritionists	795	Western herbal medicine, naturopathy	More positive than negative changes were indicated to result from government regulation for: professional status (78.6%), practice standards (73.0%), education standards (72.7%), access to research infrastructure (58.4%), post-graduate education (59.5%), access to scheduled herbs/products (55.2%), quality of herbal medicines/products (46.5%), and establishing occupational boundaries (41.3%) Uncertainty was indicated regarding the impact of regulation on practitioner income (56.1%), litigation (54.0%), patient costs (51.2%) and freedom of practice (37.6%) Negative impacts were indicated to be more likely in the area of medical influence on practice (44.3%) 44% indicated their training poorly prepared them for inter-professional communication, 22% indicated they were poorly prepared in the area of clinical training
Boon 2004 [53]	Canada (Toronto, Ontario)	Qualitative Focus groups	Acupuncture/traditional Chinese medicine practitioners, homeopaths, naturopaths	20	Acupuncture/traditional Chinese medicine, homeopathy, naturopathy	Statutory regulation was identified as the goal of the professionalisation All practitioners stated their group was pursuing statutory regulation Most, but not all, felt this was an important goal for their occupation Many considered regulation would result in some form of monopoly for their practice Regulation would prevent the co-optation of their skills and knowledge, allow the achievement of social closure by establishing education and qualifications standards that would prevent co-optation by those outside the profession Attempts at closure were hampered by lack of internal cohesion, and disagreement over the content and form of education and practice standards, particularly for homeopathy and acupuncture/traditional Chinese medicine professions Fragmentation was partially due to the intra-professional diversity of practices and philosophies Some feared a loss of freedom to practise due to regulation In T&CM finding a place within the health care system, the public was one of their biggest allies Some homeopaths and naturopaths recast their work as possibly harmful in order to be eligible for regulation
Braun 2013 [74]	Australia	Quantitative Online questionnaire survey	Naturopaths, Western herbal medicine practitioners	479	Western herbal medicine, naturopathy	85% agreed practitioners should be formally registered to safeguard the public, 8% disagreed, 8% were unsure Responses indicated possible benefits of regulation were increased public safety, protection from inadequately trained practitioners within and outside their profession, increased practice standards, increased confidence and credibility of the profession and its broader integration into the health care system Some (*n* = 9) who agreed with regulation did not agree that public safety was the main issue, stating that being a member of a professional association was the same as registration so formal registration was not necessary Others (*n* = 9) expressed concern that practitioners outside their profession could regulate their profession if formal registration occurred
Canaway 2009 [71]	Australia (Melbourne)	Qualitative Semi-structured interviews Snowball sampling	Naturopaths (senior practitioners mostly in leadership roles)	7	Naturopathy	Registration was necessary to safeguard the public, raise education standards in line with public expectations, ensure transparent complaints handling, guard against unscrupulous practitioners, maintain professional and ethical standards, provide guarantees regarding practitioner education, improve relationships with medical professionals, provide access to hospitals, protect the interests of the profession such as misappropriation of practice, could promote professional unity, and limit the proliferation of profit-oriented private colleges Regulation was also necessary because it was not the job of the professional associations Potential positive impacts were increased status, legitimacy and acceptance particularly by the medical profession, opening the way for medicare rebates, accessing restricted herbs, aiding the removal of incompetent practitioners, gaining a greater share of the health care market, establishing higher minimum education standards and greater consistency in the quality and length of training Registration was unnecessary because natural therapies were safe when offered by trained practitioners, a registered profession is not necessarily a safe profession, current complaints handling was adequate, protection of title did not prevent unsafe practitioners from using different titles, it inappropriately defined the profession as unsafe, it only conferred status and bolstered self-esteem which is not its purpose, other T&CM registered professions disliked it, current self-regulation was working with most practitioners being members of professional associations which monitored standards so there was little to gain Potential negative impacts were restrictions to practice, loss of freedom to practise, standardisation of practice, could attract different types of people to the profession motivated by status, increased professional indemnity costs and registration fees that would be passed onto clients, not all practitioners may be eligible for registration which would be negative for them, uncertainty of who the profession would be answerable to, greater emphasis on scientific aspect of education and less on the holistic approach, erosion of naturopathic philosophy, and closure of colleges unable to meet the new standards
Cottingham 2015 [96]	New Zealand	Quantitative Online questionnaire survey. Convenience sampling	Naturopaths and herbal medicine practitioners	107	Herbal medicine, naturopathy	62% supported statutory registration, 18% were opposed.^a^ 82% supported registration, 75% of which were in favour of statutory registration and 25% supported voluntary registration
Cottingham 2017 [97]	New Zealand	Quantitative Online questionnaire survey. Convenience sampling	Homeopaths	47	Homeopathy	51% supported statutory registration.^a ^ 87% favoured registration, of which 59% supported statutory registration
Cottingham 2018 [82]	New Zealand	Quantitative Online questionnaire survey. Convenience sampling	Registered massage therapists	104	Massage	56% supported statutory registration.^a^ 93% supported registration, of which 67% supported statutory registration, 31% supported voluntary registration, 1% preferred other registration types (not-specified)
Ericksen-Pereira 2020 [55]	South Africa	Qualitative Emailed unstructured (open-ended) survey or face to face unstructured interviews	Naturopaths	21	Naturopathy	Registration was important to all participants because it allowed them to use the title of naturopath and practise legally Challenges of registration created impediments to establishing viable practices, including being deregistered when failing to pay on time, bureaucracy requiring further application fees and increased annual re-registering fees, the need to register within the first six months of graduating or undergo a competency assessment requiring a fee, annual registration fees regardless of whether graduates were in a financial position to establish a practice, costs of registration (around 20% of the average salary of a new graduate), being prevented from sharing clinic space or working in interdisciplinary practices with registered practitioners such as medical doctors which prevented integrative practices
Flatt 2013 [71]	New Zealand	Quantitative Postal questionnaire survey	Naturopaths and Western herbal medicine practitioners	120	Western herbal medicine, naturopathy	Over 60% agreed that change of regulatory status would have positive effects on professional status, professional relationships, integrative practice, potential health subsidies, practitioner competence, and practice and education standards Over 50% agreed that change of regulatory status would have positive effects on professional equity, shared care, conduct and discipline and continuing education Almost 60% disagreed that regulatory change would have a positive effect on association fees Just under 50% felt there would be negative effects on freedom of practice Around 50% were uncertain about the effects on income, litigation, research access, biomedical influence and career prospects There was little agreement on the impact on occupational boundaries, access and quality of medicines, patient numbers, patient well-being, and patient access
Gyasi 2017 [56]	Ghana (Ashanti Region)	Qualitative In-depth interviews	Traditional healers	7	Traditional healing	Despite being available, most practitioners were not registered and practised without any regulatory oversight Most registered practitioners were located in urban areas, where there was greater competition and need to uphold ethical and quality standards Registered practitioners thought registration was important to assure consumers of the quality of medicines sold and for the preparation of herbal prescriptions Reasons for not registering include difficulties such as having to travel to town to register, the time and cost involved, the complexity and stress, and the lack of information about how to register Non-registered practitioners were ready to register given the opportunity
Ijaz 2015 [50]	Canada (Ontario)	Qualitative Online and postal questionnaire survey analysis of open-ended question	Chinese medicine practitioners, homeopaths, naturopaths	688	Acupuncture/Chinese medicine, homeopathy, naturopathy	The majority supported regulation but 33% of Chinese medicine practitioners, 44% of homeopaths and 48% of naturopaths were concerned about regulation Concerns related to the way regulation was being implemented, and whether the regulating body overseeing the implementation was representative of the profession A number were concerned about unwanted financial and administrative burdens on practitioners, and costs being passed onto patients Concerns regarding regulation were that regulation would reduce or restrict practice scopes, or would not expand adequately to allow for diagnostic rights and biomedical testing, inter-occupational overlapping practice scopes, co-optation of practice, including co-optation from other T&CM professions (e.g. between homeopaths and naturopaths), inappropriate or unfair registration standards, e.g. how regulation would assess practitioner qualifications, grandparenting provisions, and language proficiency requirements, some practitioners may be inappropriately excluded from registration, and that regulatory changes threatened underlying paradigmatic foundations of practice Several were concerned that practice would become 'medicalized', and that regulators did not adequately take into account the 'culture and tradition' of practices Some considered that training of some practitioners was inadequate Some stated that enforcing standards would likely improve quality of patient care by raising practitioner level of treatment, and increased biomedical training would improve competency and enhance credibility A few felt there should be no grandparenting, rather, there should be an exam in order to have a legal license A small number were concerned about the use of public safety messages to increase regulatory control over low-risk activities
Malhotra 2020 [57]	Australia	Qualitative Semi-structured telephone interviews	Naturopaths	20	Naturopathy	A primary barrier to implementing integrated care models was the lack of acknowledgment from conventional medical practitioners Conventional doctors often had concerns regarding the efficacy of naturopathic treatments The current self-regulation model and absence of statutory regulation was seen as a barrier to legitimising the profession The lack of a well-defined curriculum and standard training competencies framework was considered a barrier to being acknowledged by conventional medical practitioners as a legitimate profession
Morin 2017 [88]	Canada (Quebec)	Quantitative Postal questionnaire survey	Osteopaths	297	Osteopathy	62% would be moderately/greatly influenced by government regulation and the establishment of university-based programs, 38% would not be very influenced/influenced by regulation Practitioners indicated regulation would promote collaboration, 97.2% agreeing that collaborating with physicians was slightly/quite/very important
Parker 2013 [78]	Canada (Ontario)	Quantitative Cross-sectional online questionnaire survey	Homeopaths	329	Homeopathy	Most supported regulation (mean agreement score 3.9 (SD 1.2), 5 point Likert scale, *n* = 273) Most felt regulation would: improve credibility with the public (4.3 (SD 1.0), *n* = 275) and other health care professionals (4.1 (SD 1.1), *n* = 274), benefit the public (4.0 (SD 1.2), n = 275), improve quality of patient care (3.8 (SD 1.2), *n* = 275), and benefit homeopaths (3.6 (SD1.3), *n* = 273) There was less certainty regarding the impact on practice (2.9 (1.4), *n* = 270) 70% intended to apply for registration but 35.9% appeared ineligible due to not meeting educational or grandparenting requirements Those that appeared both eligible and ineligible for registration generally supported regulation Those potentially ineligible were older, had been practising for longer, were more likely to work in a home based office, appeared to spend less time with patients on initial visit, and were less likely to hold a college/university degree
Smith 2015 [58]	New Zealand	Qualitative Semi-structured interviews	Massage educators, therapists, and students	20	Massage	Practitioners supported both government regulation and regulation by the professional association. Some were opposed to compulsory statutory regulation Regulation was viewed as a useful step to move the profession towards legitimation and professional recognition Both statutory and self-regulation were identified as mechanisms to control practice, establish a scope of practice, achieve recognition, access government funding, achieve consistency, facilitate professionalism, and establish standard education levels Practitioners stressed the need for clear scopes of practice Education required a cohesive set of standards across education providers Issues regarding regulation were cost, being valued as a profession, government regulation would establish standards of education, professional association has standards and rules Some thought degree level education should be the benchmark, others thought there was room for both diploma and degree levels as long as scopes were clear
Steel 2020 [60]	Australia (Queensland)	Qualitative Semi-structured interviews	Complementary medicine practitioners specialising in maternity care (acupuncturists, doulas, chiropractors, massage therapists, naturopaths, osteopaths)	23	Acupuncture, chiropractic, doula, massage, naturopathy, osteopathy	Regulation was needed to gain increased respect from other professions and because of concerns about unethical financial practice such as profiteering by practitioners Education standards were a concern for some participants due to a lack of consistency across institutions and qualifications Education standards was also perceived by some practitioners to impact the ability of other health professions to work alongside them, some practitioners perceiving that inconsistency in training created a negative perception of their profession among conventional providers Often there was disagreement about the impact of regulation, however there was agreement from both regulated and unregulated professions that regulation was tied to improved training standards Regulation was not always viewed favourably by participants due to the perception of practice limits being imposed Some suggested the absence of regulation created difficulties in referring peers to appropriate colleges of education
Tsai 2008 [92]	Taiwan (Taipei)	Quantitative Print-based cross-sectional questionnaire survey	Folk medicine practitioners	400	Ba guan, gua sha, reflexology, tuina	Around one half of all respondents agreed that statutory regulation was necessary for all practices 64%-85% agreed credentialling of folk medicine practitioners was necessary 65%-82% agreed practitioners should receive formal education/training Support for regulation: 56.9% of city versus 50.8% of country ba guan practitioners (*p* = 0.26) 52.8%/42.1% city/country gua sha practitioners (*p* = 0.04) 53.8%/42.7% city/country reflexology practitioners (p = 0.04) 58.4%/58.5% city/country tuina practitioners (*p* = 1.0) Opposition to regulation: 43.1%/49.2% city/country ba guan practitioners (*p* = 0.26) 47.2%/57.9% city/country gua sha practitioners (*p* = 0.04) 46.2%/57.3% city/country reflexology practitioners (*p* = 0.04) 41.6%/41.5% of city/country tuina practitioners (*p* = 1.0)
Wardle 2013 [69]	Australia (Darling Downs)	Qualitative Semi-structured interviews	Naturopaths	20	Naturopathy	Regulation was considered positive by all but one practitioner Regulation was seen by many practitioners as the solution to many of the problems of the profession The primary reason for supporting regulation was to rid the profession of unethical, bogus, or fraudulent individuals who were practising without the required qualifications Regulation would overcome the challenges of increasing external influences, internal division/fragmentation, professional acceptance, problems in education of practitioners, and co-optation by undertrained/fraudulent/practitioners that devalued the profession
Yu 2015 [100]	Korea	Quantitative Email, postal and print-based questionnaire survey	Acupuncturists, alternative therapists, chiropractors, clinical art therapists, feet massage therapists, laughter therapists, Qi gong therapists and trainees of traditional Chinese medicine	62	Acupuncture, alternative medicine, chiropractic, clinical art, feet massage, laughter, Qi gong, trainees of traditional Chinese medicine	Across all provider groups, medical and T&CM professionals, 32.8% agreed legislation to govern providers was preferable, 25.0% agreed with qualifications/accreditation, 13.6% agreed with making/evaluating standards 48.4% agreed that a college/university qualification was necessary for gaining qualifications, 22.6% agreed with taking and passing a government administered examination 53.3% agreed legislation and national control for management of qualifications was preferable, 29.0% agreed that a national examination was preferable
**Conventional medicine practitioners (*n* = 23)**						
Al Mansour 2015 [95]	Saudi Arabia (Majmaah City)	Quantitative Cross-sectional Print-based questionnaire survey	Medical students	65	Complementary and alternative medicine	Prior to T&CM training 38.5% agreed the unavailability of credentialled practitioners was a barrier to their use in Western medical settings, increasing to 70.8% following training (p = 0.0006)
Barnes 2018 [61]	New Zealand	Qualitative Semi-structured telephone interviews	Practising community pharmacists	27	Complementary medicine	Regulation of practitioners such as herbalists and naturopaths was important, although there were mixed views about whether governance should be statutory or self-regulation Some suggested that access to certain medicinal plant preparations should only be through registered CMs practitioners, such as herbalists Some framed the need for regulation in terms of providing recognition and validation of practitioner expertise
Cavaco 2017 [72]	Portugal (Lisbon and Porto)	Qualitative Semi-structured interviews	Community pharmacists	6	Homeopathic dispensing by pharmacists/non-pharmacists	There were no restrictions regarding dispensing homeopathic medicines in pharmacies Participants commonly mentioned the need for regulation of homeopathic practitioners, particularly in relation to dispensing by non-pharmacists Improved legislation would benefit prescribers, increase legal protection, and improve social recognition
Chaterji 2007 [81]	USA (Washington)	Quantitative Print-based questionnaire survey	Medical students	266	Acupuncture, aromatherapy, biofeedback, chiropractic, herbal medicine, homeopathy, hypnosis/guided imagery, magnets, massage, meditation, music, nutritional supplements, prayer/spiritual healing, rolfing, therapeutic touch	71.8% agreed that the lack of credentialled providers was a barrier to the use of practices in Western medical settings
Cohen 2005 [101]	Australia	Quantitative. National postal questionnaire survey	General practitioners	579	Acupuncture, aromatherapy, Chinese herbal medicine, chiropractic, herbal medicine, homeopathy, hypnosis, massage, meditation, naturopathy, osteopathy, reflexology, spiritual healing (e.g. reiki), vitamin and mineral therapy, yoga	The practices that required regulation were chiropractic (88% agreed), acupuncture (87%), Chinese herbal medicine (80%), hypnosis (79%), herbal medicine (77%), naturopathy (73%), osteopathy (72%), homeopathy (66%), vitamin and mineral therapy (66%), yoga (49%), meditation (44%), spiritual healing (e.g. reiki) (36%), aromatherapy (34%), massage (33%), and reflexology (28%) The practices that government should not regulate were: yoga (49% agreed), meditation (44%), spiritual healing eg. reiki (36%), aromatherapy (34%), massage (33%), reflexology (28%), vitamin and mineral therapy (18%), homeopathy (13%), naturopathy (11%), osteopathy (10%), hypnosis (9%), herbal medicine (8%), Chinese herbal medicine (7%), acupuncture (6%), and chiropractic (4%) Areas of greatest concern were incorrect/inadequate/delayed diagnosis, interactions between complementary medicines and pharmaceuticals, and patients not disclosing T&CM use to their doctors Some commented that complementary therapies caused no harm in the hands of appropriately trained practitioners Many GPs indicated that therapies that did not involve medicines (such as yoga, meditation, and spiritual healing) did not require regulation, whereas others that had potential to delay diagnosis or interact with conventional medication (such as herbal medicine or naturopathy) should be regulated Some suggested self-regulation (e.g. industry accreditation) was more appropriate for some therapies than government regulation
Dooley 2010 [75]	Australia (Gold Coast, Melbourne, and Wagga Wagga)	Quantitative. Postal, online, and print-based questionnaire survey	Pharmacy assistants	107	Western herbal medicine, naturopathy	75% strongly agreed/agreed naturopaths should be formally registered to safeguard the public, 2% disagreed, 22% were unsure
Flower 2015 [62]	UK	Qualitative Semi-structured face to face and telephone interviews	General practitioners	15	Herbal medicine	Practitioner regulation and proper quality control procedures were key factors that were required before herbal treatments could be recommended Many were open to the use of herbal medicines when conventional treatment had failed Concerns were expressed about the lack of quality assurance of herbal products, potential adulteration with pharmaceuticals, and possible interactions between herbs and drugs The training, lack of regulation or licensing, and the level of medical knowledge of herbal practitioners, were additional sources of uncertainty
Hall 2000 [103]	Australia (Perth)	Quantitative Cross-sectional postal questionnaire survey	General practitioners	282	Acupuncture, aromatherapy, herbal medicine, homeopathy, hypnosis, massage, naturopathy, meditation, spinal manipulation, yoga	32.2% did not favour referral to practitioners citing lack of government regulation and training standards GPs were most likely to refer patients to acupuncture (75.0% of respondents), massage (62.0%), meditation (53.0%), and yoga (42.0%) Fewer than 8% would refer patients to aromatherapy, herbal medicine, homeopathy, or naturopathy
Harris 2006 [84]	USA (Minnesota)	Quantitative Print-based questionnaire survey	College of Pharmacy faculty department members, and pharmacy students	94	Acupuncture, aromatherapy, bioelectromagnetic therapies, biofeedback, chiropractic, herbal medicine, homeopathy, hypnosis/guided imagery, massage, music, nutritional supplements, prayer/spiritual healing, meditation, rolfing, therapeutic/healing touch	58% of faculty members and 80% of students (*p* = 0.074) agreed the unavailability of credentialled providers was a barrier to the use of T&CM practices 74% of faculty members and 53% of students indicated chiropractic a mainstream health care practice 53% of students indicated nutritional supplements mainstream practice No other practice achieved > 50% agreement on being a mainstream practice
James 2020 [59]	Sierra Leone (Freetown in Western Area, Bo district in Southern Region, Kenema district in Eastern Region, Bombali district in Northern Region)	Qualitative Semi-structured interviews	Doctors, nurses, and community health officers	15	Traditional and complementary medicine	The prerequisites for health care integration included training of T&CM practitioners on what diseases to treat and what to refer, regulation of practices, and public education to seek care from licensed practitioners only T&CM practitioners should be adequately educated in basic medical training Collaboration with some T&CM practices, such as herbal medicine, which were perceived to cause serious adverse effects, should not be permitted
Jarvis 2015 [61]	England (Liverpool and Manchester)	Qualitative Semi-structured telephone interviews	General practitioners	19	Complementary and alternative medicine	Practitioners should be professionally regulated GPs were significant concerned about herbal remedies (e.g. causing interactions with pharmaceuticals) Having greater confidence in the robustness of practitioner training and regulatory procedures enables GPs to have greater confidence in endorsing practices and referring patients to practitioners
Langworthy^b^ 2000 [85]	Netherlands	Quantitative Postal questionnaire survey	Osteopaths, manual therapists, physiotherapists	227	Chiropractic	59% of osteopaths (*n* = 49), 24% of manual therapists (*n* = 46), and 15% of physiotherapists (*n* = 132) agreed chiropractors should be 'politically recognized' through statutory registration 13% of manual therapists and 3% of physiotherapists agreed chiropractors should not be 'politically recognized' through statutory registration 67% of manual therapists agreed chiropractic was in competition with manual therapy 22% of osteopaths agreed chiropractic was in competition with osteopathy 19% of physiotherapists agreed chiropractic was in competition with physiotherapy
Livingston 2010 [86]	Australia (Sydney)	Quantitative Postal questionnaire survey	General practitioners	288	Herbal medicine, naturopathy	91% strongly supported a national register for naturopaths and herbalists, requiring qualifications for listed members to be licensed 84.0% agreed that herbal therapies should be regulated in the same way as pharmaceuticals, 7.3% disagreed
Montbriand 2000 [87]	Canada (Saskatchewan)	Quantitative Postal questionnaire survey	Nurses, pharmacists, physicians	153	Alternative therapies	Across all three professional groups 69% agreed with regulation, 10% did not, 21% were undecided 82% of pharmacists (*n* = 49) agreed T&CM should be regulated or have government controls, 10% did not agree, 8% did not know Pharmacists were alarmed at the number of herbal and homeopathic products entering the market without standards, regulations and proper labelling 69% of physicians (*n* = 52) agreed T&CM should be regulated or have government controls, 15% did not agree, 15% did not know Physicians felt practices with potentially harmful side-effects should be regulated, but were concerned about the impracticality of controls and whether effective regulation was possible 56% of nurses (*n* = 52) agreed T&CM should be regulated or have government controls, 6% did not agree, 38% did not know Nurses focussed comments on practitioners, expressing the need for self- or government regulation and certification to practice
Morin 2017 [88]	Canada (Quebec)	Quantitative Postal questionnaire survey	Physicians	266	Osteopathy	Physicians supported the regulation of osteopathy and indicated it would promote collaboration 72% would be greatly/moderately influenced by government regulation and the establishment of university-based programs, 28% would not be influenced/very influenced by regulation Physicians indicated regulation would promote collaboration, 85.3% agreeing that collaborating with osteopaths was slightly/quite/very important
Poreddi 2016 [104]	India (Bangalore)	Quantitative Cross-sectional Print-based questionnaire survey	Student nurses	122	Acupuncture, Ayurveda, aromatherapy, biofeedback, chiropractic, herbal medicine, homeopathy, hypnosis, naturopathy, reflexology, spiritual healing	81.1% agreed that the unavailability of credentialled providers was a barrier to the use of T&CM, 18.9% did not agree
Poynton 2006 [98]	New Zealand	Quantitative. Nationwide cross-sectional postal questionnaire survey	General practitioners	300	Acupuncture, aromatherapy, traditional Chinese medicine, chiropractic, herbal medicine, homeopathy, hypnosis, traditional Māori medicine, naturopathy, osteopathy, traditional Pacific Island medicine, reflexology, spiritual healing	Less than 12.3% of GPs referred patients to aromatherapy, herbal medicine, naturopathy and traditional Chinese medicine, traditional Māori medicine, and traditional Pacific Island medicine, compared to greater than 70.0% referral rate to acupuncture, chiropractic, and osteopathy The most common reasons for not referring patients were lack of evidence (88%, *n* = 264), lack of regulation (78%, *n* = 234), and financial cost to patients (50.3%, *n* = 151) Other reasons for non-referral include concerns about exploitation of vulnerable patients and the risk of adverse effects or harm
Price 2004 [89]	UK	Quantitative. Population-based postal questionnaire survey	Members of the British Medical Acupuncture Society (including general practitioners (75% of respondents), hospital doctors and other health professionals)	1112	Acupuncture	56% favoured of some form of regulatory governing body for practitioners, 27% were undecided
Semple 2006 [90]	Australia (South Australia, Queensland, and Victoria)	Quantitative Postal questionnaire survey	Practising pharmacists	211	Complementary and alternative medicine practices dispensing vitamins and minerals, flower remedies, herbal products and other dietary supplements, homeopathic products, tissue salts	Pharmacists agreed the current level of regulation of practitioners was a barrier to information provision about T&CM to consumers. One way of overcoming this was indicated to be better regulation of practitioners
Taylor 2003 [91]	New Zealand (Wanganui)	Quantitative Postal questionnaire survey	General practitioners	25	Acupuncture, aromatherapy, chiropractic, colour therapy, homeopathy, hypnosis, iridology, reflexology, Rongoā Māori	84% indicated they would like to see better regulation of therapies 96% indicated concern about the safety of therapies
Tiralongo 2010 [99]	Australia	Quantitative. National postal questionnaire survey	Community, hospital, consultant, industry, academia, public service, and army pharmacists	583	Herbal medicine, naturopathy	92% strongly agreed/agreed that practitioners should be formally registered to safeguard the public, 3% disagreed/strongly disagreed, 5% were unsure
Wardle 2018 [51]	Australia (Non-metropolitan New South Wales)	Qualitative. Population-based online and postal questionnaire survey analysis of open-ended question	General practitioners	152	Complementary medicine	Risk was a major theme in many of the responses, with both direct (e.g. drug-herb interaction, adverse events), and indirect risks (e.g. delayed diagnosis, exploitation) identified Most held that risks were maximised due to the variability of standards, practices and treatments which was related in large part to the regulatory vacuum Practitioner or product variability or potential monopolisation of care by providers were highlighted as major issues that increased potential risks to patients. For many GPs, it was this risk, rather than risk of ineffective therapies, which was of most concern Both supporters and detractors of T&CM were concerned about the lack of regulation Providers practised in a regulatory vacuum and were considered to be practising without any regulatory oversight and therefore without restrictions
Yu^c^ 2015 [100]	Korea	Quantitative Email, postal and print-based questionnaire survey	Doctors, nurses, Oriental (Korean) medical doctors as medical professionals	19	Acupuncture, alternative medicine, chiropractic, clinical art, feet massage, laughter, Qi gong, trainees of traditional Chinese medicine	Across all provider groups, medical and T&CM professionals, 32.8% agreed legislation to govern providers was preferable, 25.0% agreed with qualifications/accreditation, 13.6% agreed with making/evaluating standards 31.6% agreed government administered examinations were the most important for gaining T&CM qualifications, 15.8% agreed that qualifications from a certified institute, e.g. college/university was preferable 29.4% agreed a national examination for managing qualifications was preferable, 23.6% agreed with legislation and national control as the preferred option
**Professional associations (*n* = 6)**						
Clarke 2004 [54]	UK	Qualitative. Document analysis	T&CM professional associations representing aromatherapy, Chinese herbal medicine, chiropractic, crystal healing, feng shui, 'lay' homeopathy, medical homeopathy, osteopathy and radionics	9	Aromatherapy, Chinese herbal medicine, chiropractic, crystal healing, feng shui, 'lay' homeopathy, medical homeopathy, osteopathy, radionics	Most associations promoted the need for tighter regulation Professionalisation strategies were necessary to eliminate unprofessional conduct, ensure autonomy of practice and promote legitimacy of practitioners The Chinese herbalists associations and chiropractic associations were most concerned with professionalisation The Chinese herbalists associations were committed to statutory self-regulation arguing this was the best option to ensure professional recognition, establish protection of title and give authority to use scheduled herbs Leaving regulation unaddressed would risked imposition of government controls Regulation provided greater protection for patients, increased credibility, raised the visibility of herbal medicine and protected practice autonomy Challenges to regulation was gaining consensus between associations Concerns included dilution of philosophical traditions and practice standardisation
Gilmour 2002 [63]	Canada (Ontario)	Qualitative. Unstructured interviews	Leaders in the professions of acupuncture/traditional Chinese medicine, homeopathy, and naturopathy	24	Acupuncture/traditional Chinese medicine, homeopathy, naturopathy	Statutory self-regulation was seen as key to full professionalisation by all groups. They desired the protection of statutory regulation which included status and legitimacy, acknowledgement of skills and qualifications, potential integration into the health care system, restricted use of designated titles to registered members, acceptance of practices by private insurers, provision of a defined scope of practice, to assure consumers of the quality of training and protection against those who did not meet the standards All groups expressed the hope that regulation would lead to the establishment and enforcement of standards of practice but were unable to accomplish this due to internal disagreements, division, lack of cohesion, and intra-professional competition, all of which hindered regulation attempts Only the naturopaths saw the need for appropriate research to support the push for regulation, the two remaining groups were content to rely on historical evidence
Kelner 2004 [67]	Canada (Ontario)	Qualitative Semi-structured interviews, and published policy material	Directors or presidents of conventional professional associations of medicine, nursing, physiotherapy, clinical nutrition and public health, and federal/provincial statutory governing bodies of the professions of clinical nutrition, medicine, nursing, physiotherapy, and public health	10	Acupuncture/traditional Chinese medicine, chiropractic, homeopathy, naturopathy, reiki	Most were unsympathetic to the professionalisation of T&CM groups Achieving statutory self-regulation was important for professionalisation but had to be earned. There was a reluctance to encourage T&CM groups to gain regulation They argued that unless therapies had a body of knowledge based on scientific evidence and a method of delivering care in an objective, standardised way, it was unsafe to allow practitioners to treat patients Higher standards of evidence were essential to gain formal recognition and a place in the health care system In order for T&CM practitioners to be credentialled, their therapies and practices would need to be evidence-based Nursing representatives more often emphasised the need for public safety and protection as a rationale for regulating T&CM providers, rather than a need for evidence Allied health professions felt strongly that regulation should not be granted until there was scientific evidence that therapies were safe and effective and scopes of practice were suitable Regulation would promote referrals, especially from the nursing profession The issue of scope of practice evoked tensions and concerns about maintaining jurisdictional boundaries and protecting turf Leaders proposed several ways to block or control integration; co-optation, physicians as gatekeepers, and opposing government funding for T&CM
Kelner 2006 [68]	Canada (Ontario)	Qualitative Semi-structured interviews, and documentary material	Leaders in the professions of chiropractic and homeopathy	16	Chiropractic, homeopathy	Common professionalisation strategies were used by both professions, i.e. improving education quality, raising practice standards, developing research capacity, and increasing group cohesion There was insufficient support from government for professionalisation efforts Achieving statutory self-regulation motivated chiropractors to pursue professionalisation strategies Regulation gave authority to enforce practice standards and monitor ethical misconduct, but did not improve professional harmony As an unregulated profession, homeopathy had scarce resources, and greater difficulty in maintaining clinical standards and sanctioning unethical practitioners because of inadequate monitoring. They also had greater internal division which impeded professionalisation efforts Some homeopathy leaders believed regulation would promote cohesion and raise practice standards
Lin 2005 (Section 6, McCabe) [52] (Summarised in [105])	Australia	Quantitative Postal questionnaire survey	T&CM professional associations of naturopathy and Western herbal medicine	11	Western herbal medicine, naturopathy	36% of associations supported statutory regulation, 36% supported the existing self-regulatory model, 27% wanted stronger regulation (unspecified) 64% of associations were negative about the existing self-regulatory model, in part because it had not produced a national, consistent, or effective regulatory system The need to protect the professions and practices was seen as strong motivators by supporters and opposers of statutory regulation There was no uniform minimum standard of education, and concerns about inadequately trained or incompetent practitioners Some supporters of statutory regulation argued that this status, and with it raised standard of education, was essential to gain access to currently scheduled natural medicines Others believed statutory regulation could introduce unacceptable restrictions on practice, and care should be taken that the regulatory effect on education, particularly increasing medicalisation of naturopathy, did not diminish the founding philosophies There was potential for conflict of interest if professional associations were linked with private education providers, whose commercial interests might not be served by the requirement to raise standards of education
Welsh^d^ 2004 [70]	Canada (Ontario)	Qualitative Semi-structured interviews	T&CM senior leaders of the associations of traditional Chinese medicine/acupuncture, homeopathy, and naturopathy	24	Acupuncture/traditional Chinese medicine, homeopathy, naturopathy	Strategies employed to achieve statutory self-regulation were improving education and practice standards, engaging in peer-reviewed research, and increasing group cohesion The inclusion of medical science was considered the basis of distinguishing between 'science' and 'non-science' and who should practise and who should not. All groups attempted to demarcate knowledge claims from competitors, and they all engaged in boundary work. The diversity of knowledge claims made uniform standards difficult to achieve Leaders looked forward to the raised minimum education standards that regulation would impose but differing educational standards between homeopathy schools impeded the setting of uniform standards. High standards were seen as important to protect the public Some saw the need for more clinical and peer-reviewed research to support regulation claims but debated what research was required Challenges included no intra-professional agreement on the best standards to follow, which knowledges to codify or what kind of research should be conducted Some considered encouraging more cohesion through conflict resolution to successfully deal with government and achieve regulatory status
**Education providers (*n* = 2)**						
Lin 2005 (Section 5, McCabe) [52] (Summarised in [80])	Australia	Quantitative Postal questionnaire survey	Education providers of undergraduate and post-graduate courses	19	Western herbal medicine, naturopathy	Overall 53% preferred statutory regulation of practitioners 64% of private providers (*n* = 14) supported government involvement in regulation, 36% supported statutory regulation, 28.5% supported co-regulation, 7% supported continued self-regulation 100% of universities (*n* = 5) supported statutory regulation A degree level of education was essential 'for the good of the profession’, and to provide university pathways, though only 45% of providers supported a bachelor's degree as the minimum requirement A major concern of private providers was that moving courses into the university sector might result in a loss of traditional holistic philosophies and perspectives There was about ongoing conflict within the professions over regulation and education Major concerns were lack of agreement on the most appropriate model of regulation and minimum educational standards, the commercialisation of education resulting in lowered standards, lack of democratic processes and transparent policies in some professional associations, and the need for standards to be set by an independent body
Welsh^d^ 2004 [70]	Canada (Ontario)	Qualitative Semi-structured interviews	T&CM senior leaders of the major schools of traditional Chinese medicine/acupuncture, homeopathy, and naturopathy	24	Acupuncture/traditional Chinese medicine, homeopathy, naturopathy	Strategies employed to achieve statutory self-regulation were improving education and practice standards, engaging in peer-reviewed research, and increasing group cohesion The inclusion of medical science was considered the basis of distinguishing between 'science' and 'non-science' and who should practise and who should not. All groups attempted to demarcate knowledge claims from competitors, and they all engaged in boundary work. The diversity of knowledge claims made uniform standards difficult to achieve Leaders looked forward to the raised minimum education standards that regulation would impose but differing educational standards between homeopathy schools impeded the setting of uniform standards. High standards were seen as important to protect the public Some saw the need for more clinical and peer-reviewed research to support regulation claims but debated what research was required Challenges included no intra-professional agreement on the best standards to follow, which knowledges to codify or what kind of research should be conducted Some considered encouraging more cohesion through conflict resolution to successfully deal with government and achieve regulatory status
**Policy-makers (*n* = 2)**						
Kelly 2005 [65]	Canada (Alberta, British Columbia)	Qualitative Semi-structured interviews	Regional health policy-makers	10	Acupuncture, chiropractic, herbal medicine, homeopathy, hypnosis, massage	Policy-makers held a positive view for the integration of T&CM at the clinical and primary care levels of practice Public safety was an important concern Policy-makers supported the movement towards integrative health services, but emphasised that the issues of evidence-based T&CM research, standards of accreditation and training for T&CM practitioners, as well as the issue of who pays, needed to be addressed to ensure the improved health and well-being of the public CAM credibility and the potential for integration suffered from a lack of evidence demonstrating beneficial outcomes More rigorous training and licensing of T&CM practitioners would contribute positively to movement towards integration Poor and uneven accreditation of T&CM practitioners was a significant barrier More thorough and consistent accreditation procedures for T&CM practitioners would increase legitimacy in the eyes of the conventional healthcare system, the government and the general public Some identified the need for medical training in the T&CM curricula and viewed this step as the 'point of real leverage' for T&CM integration A few felt that integration was hampered by potential boundary disputes between conventional and T&CM practitioners
Kelner 2004 [66]	Canada (Ontario)	Qualitative Semi-structured interviews	Federal, provincial, and municipal government health department officials	10	Acupuncture/traditional Chinese medicine, chiropractic, homeopathy, naturopathy, reiki	Most comments were focussed on acupuncture/traditional Chinese medicine, chiropractic, and naturopathy The role of the state was to fulfil their mandate to protect the public while responding to consumer demand for T&CM services Statutory self-regulation was regarded as a mechanism for ensuring public safety, creating accountability for practitioners Regulation was regarded as a bargain that confers legitimacy, social inclusion, and socioeconomic status in exchange for constraints that protected the public interest Most said they could foresee a legitimate place for T&CM groups within the health care system, but it was essential to establish evidence of effectiveness and safety, standards of training, credentialling, effective control over practice, and clear accountability Practitioners would achieve better acceptance from conventional medicine and government if they had scientific evidence of efficacy and safety Governments hesitated to endorse the 'legitimation' of T&CM due to concerns about the costs of health care that they feared T&CM groups would make demands on. Cost of health care was seen as a barrier to integrating T&CM into mainstream medicine A clear and appropriate scope of practice was essential for T&CM groups to gain a 'legitimate' place in the system. An example was naturopathy with a broad scope that overlaps with other specialities, making it difficult to achieve social closure and hampered efforts to make jurisdictional claims. It could also infringe on the practices of medical professionals and hinder legitimation and integration of T&CM Disorganisation, internal conflicts and tensions, and fragmentation of some T&CM groups made engagement with government difficult and hampered integration into the health care system

^a ^This percentage has been calculated by the first author using raw data supplied in the published article

^b ^Physiotherapists, manual therapists and osteopaths were considered conventional medical practitioners in the country of research

^c ^Oriental medical doctors were considered conventional medicine professionals in the country of research

^d ^This study examined both professional association and education provider stakeholders

### Attitudes to regulation

Fifteen of 24 quantitative studies reported greater than 60% support for the regulation of T&CM practices. Across all stakeholder groups there was between 15% and 95% (median 61%) support for, and 1% to 57% (median 14%) opposition to the regulation of various T&CM professions.

Between 71% and 95% of consumers (median 86%) were supportive of T&CM provider regulation [76, 79, 83, 91, 93, 94, 102], and 1% to 5% (median 2%) were opposed [76, 79, 102].

T&CM practitioner support for regulation was between 33% and 85% (median 54%) [74, 82, 92, 96, 97, 100], while 8% to 58% (median 43%) opposed regulation [74, 82, 92, 96]. Notably, the main opposition to regulation came from outlier results in one study of Taiwanese folk medicine practitioners of ba guan, gua sha, reflexology, and tuina whose disapproval of regulation ranged from 42% to 58% [92]. Removing these outlier data points from the results shows median T&CM practitioner opposition to regulation was 25% [74, 82, 96, 100].

Some 15% to 92% (median 66%) of conventional medicine providers supported [75, 85–87, 89, 91, 99–101], and 2% to 49% (median 11%) opposed [75, 85–87, 99] T&CM regulation.

Two-thirds of professional association studies [54, 63, 68, 70] reported endorsement for regulation, the only quantitative study finding that 36% of Australian associations supported regulation, 36% preferred self-regulation, and 27% called for a stronger, unspecified model of regulation [52]. Canadian T&CM associations [63, 68, 70] and education providers [70] reported regulation was necessary for the professionalisation of T&CM practices. In contrast, only 53% of Australian education providers supported regulation, with significant differences between private (36% support) and university (100% support) sector providers [52]. Representatives of Canadian conventional medicine associations indicated reluctance for encouraging T&CM groups to pursue regulation and reported that while regulation may be important for T&CM it had to be earned through established evidence and standards [67]. Canadian policy-makers were generally supportive of T&CM professional regulation and integration into mainstream health provision [65, 66].

Consumers favoured regulation for certain T&CM professions, indicating it should be the same as for medical practitioners in half of all consumer studies [79, 83, 91, 93]. In decreasing order of highest reported percentage consumers in Australia, New Zealand, and UK supported the regulation of Ayurveda, naturopathy (90%) [79], herbal medicine (90%) [76, 79], homeopathy, osteopathy, reflexology (90%) [76], Chinese medicine (76%-90%) [79, 93, 94], acupuncture (71%-90%) [76, 91, 94], aromatherapy, chiropractic, hypnotherapy (71%-90%) [76, 91], and Rongoā Māori (71%) [91].

Conventional medicine practitioners also preferred certain T&CM professions to be regulated. Studies from Australia, Netherlands, and UK reported provider support for regulation of Western herbal medicine (77%-92%) [86, 99, 101], naturopathy (73%-92%) [75, 86, 99, 101], chiropractic (15%-88%) [85, 101], acupuncture (56%-87%) [89, 101], and Chinese herbal medicine (80%) [101]. Most notable is the low support for regulating T&CM practices reported by Korean medical professionals (33%) [100], and for regulating chiropractors reported by physiotherapists (15%) and manual therapists (24%) in the Netherlands [85]. Also noteworthy in the latter study is that 19% of physiotherapists and 67% of manual therapists agreed that chiropractic was in competition with their profession [85].

### Public, practitioner and practice impacts of regulation

Thirty-three studies reported stakeholder reasons for attitudes towards regulation of T&CM practices. These attitudinal drivers were analysed inductively and summarised into three key themes of stakeholder impact; the public, T&CM practitioners, and T&CM practices. Full details of these findings can be found in Table 4.

#### Regulation and the public

Twenty studies reported stakeholder attitudes regarding the impact of regulation on the public. Studies from Australia, Canada, New Zealand, and UK reported consumer, practitioner, and policy-maker views that regulation of T&CM practices was needed to safeguard the public [65, 66, 75, 94, 99, 101] and protect patients from unqualified, incompetent or unethical T&CM practitioners [50, 52, 60, 65, 66, 69, 71, 74, 93].

Representatives of Canadian nursing associations emphasised public safety to justify their support of T&CM regulation, however allied health association representatives stated regulation should be deferred until T&CM practices established evidence of safety and efficacy [67]. Consumers and GPs stressed the potential for harm from T&CM treatments including interactions with pharmaceuticals [51, 62, 83, 91, 98, 101], as well as harm due to patient exploitation [51, 98], financial cost [98, 101], and inadequate or delayed diagnosis [51, 101]. In one study GPs reported that the risk of harm was primarily due to lack of regulation [51], while in another GPs indicated that well-trained T&CM practitioners caused little or no patient harm [101]. Conventional medicine practitioners in Sierra Leone stated that herbal medicine posed such serious risks they would not collaborate with T&CM practitioners regardless of regulatory status [59]. Some Australian naturopaths could not agree on whether regulation would improve complaints handling [71], while consumers identified the need for an official body to hear complaints because of doubts that self-regulated professional associations would adequately sanction errant members [52].

#### Regulation and T&CM practitioners

The training and qualifications of T&CM practitioners came under scrutiny from all stakeholder groups in 16 studies conducted in Australia, Canada, New Zealand, Sierra Leone, and UK. Consumers [76], conventional medicine practitioners [59, 62, 64, 103], and policy-makers [65, 66] expressed concerns regarding the training, qualifications or biomedical knowledge of T&CM providers. T&CM practitioners indicated that regulation would improve training standards, qualification standards [60, 69, 71, 73, 77], and practitioner competence [71, 77]. However, not all Australian and Canadian T&CM practitioners were positive about the likelihood of raised training standards due to perceptions of unfair or unachievable qualifications, grandparenting, or language proficiency requirements [50, 71].

Regulation was seen to confer intra-professional and inter-professional advantages to T&CM practitioners. These include enhancing status and prestige [50, 71, 73, 74, 77, 78], promoting professional acceptance and recognition [60, 69, 71, 73, 77, 78], granting legitimacy [63, 65, 66, 71], improving inter-professional relationships [71, 77], encouraging greater collaboration [88], and facilitating integration into health care systems [74, 77]. GPs perceived that training quality assurance would promote patient referral to T&CM providers [64] or conversely did not favour patient referral due to perceived inadequate training standards [103].

T&CM education providers and representatives of professional associations noted that improving educational standards was an integral part of achieving regulation and professionalisation [52, 63, 68, 70]. Canadian policy-makers stated that poor quality T&CM training and credentialling practices created barriers to achieving regulatory status [65, 66]. They indicated that T&CM could have a legitimate place in health care provided they demonstrated an evidence base and established appropriate standards [66].

#### Regulation and T&CM practice

The impact of regulation on practice standards, occupational title protection, and scopes of practice drew most comments from T&CM practitioners and professional associations in studies from Australia, Canada, New Zealand, and UK.

T&CM practitioners anticipated that practice, professional, or ethical standards would improve following regulation [71, 73, 74, 77], while GPs indicated that variations in T&CM standards, practices and treatments, due primarily to lack of regulation, increased risks to patients [51]. Representatives from T&CM professional associations and analysis of association documentation indicated that regulation and professionalisation provided the means for monitoring and mandating practice standards [68], and preventing unprofessional conduct [54]. In contrast, New Zealand massage therapists regarded benefits such as practitioner monitoring, education standardisation, and professionalisation were readily provided under both self-regulatory and statutory frameworks [68]. Similarly, some Australian naturopaths and herbalists argued that regulation was unnecessary because of membership of professional associations [71, 74] that maintained and monitored practice standards [71].

A key benefit of regulation according to some T&CM practitioners [55] and professional association representatives [54, 63] was the provision of legal protection of T&CM occupational titles. Though some T&CM practitioners were divided on the issue [77], and others were uncertain whether regulation could resolve the problem of overlapping practice scopes [50], regulation was regarded by some T&CM practitioners [73] and association representatives [63] as facilitating the establishment of scopes of practice or practice boundaries. For conventional medicine association representatives the prospect of a T&CM scope of practice prompted concerns over jurisdictional boundary infringements, including limiting scope for T&CM integration [67]. This view was shared by Canadian policy-makers who stated that such boundary infringements on medical practitioner territory could stymy attempts at ‘legitimation’ and integration [65, 66]. T&CM regulation was seen by policy-makers as a contractual bargain in return for T&CM accepting practice restrictions aimed at protecting the public [66]. However, the possibility of practice restrictions arising from regulation concerned some Australian consumers [52], Australian T&CM professional associations [52], and Australian, Canadian, and New Zealand T&CM practitioners [50, 53, 71, 73, 77].

Other issues perceived by T&CM providers were related to undue biomedical influence over practice or extra-professional regulatory oversight [50, 71, 74, 77]. Some practitioners indicated this could negatively affect practice [73], others considered such influence would be overcome through regulation by allowing greater autonomy [69], a position also taken by some professional association representatives [54].

A related issue concerns practice misappropriation by untrained/undertrained practitioners from other branches of health care. While T&CM practitioners perceived that regulation could prevent practice misappropriation [53, 69, 71], conventional medicine associations proposed co-optation as a way to maintain control of integration of T&CM practices by conventional medical practitioners rather than non-medical T&CM providers [67]. T&CM practitioners [50, 71] and professional association representatives [52, 54] were concerned that external influences may lead to the diminution of traditional practice philosophies, as were consumers who lamented the potential loss of an intuitive practitioner approach due to regulation [52].

## Discussion

This review provides the first known systematic examination of the contemporary empirical literature regarding stakeholder attitudes to the regulation of T&CM professions.

Of the 60 identified studies, six sought opinions from professional associations in Australia, Canada, and UK, two studies from Australia and Canada investigated education providers, and only two, both from Canada, canvassed the perceptions of policy-makers. The largely positive views of these stakeholders are tempered by the limited available research. The lack of research from many countries where T&CM is practised also limits the international generalisability of the findings. In particular, the views of policy-makers outside of Canada are yet to be determined and, consistent with other jurisdictional differences, may well be dissimilar to that seen in the present review. Scholars and commentators have long recognised the crucial role of policy-makers and policymaking in formally recognising T&CM professions. Policy-makers reported their priority of upholding the public interest while outlining a roadmap for regulation and integration. These were considered inducements in exchange for practice restrictions, which some T&CM practitioners and professional associations considered unacceptable. Further research focussed explicitly on these stakeholder groups, particularly those of policy-makers, is clearly needed to inform decisions regarding implementation of the WHO Traditional Medicine Strategy recommendations [9].

Another key finding from the review is that, consistent with regulatory policy provisions, the main focus of stakeholders outside of T&CM professions was on public protection and raising inadequate training and practice standards. While these views were dominant among T&CM practitioners as well, attention was also directed towards the professional benefits and disbenefits of regulation, suggesting that better communication regarding the purpose of regulation is needed. Previous research examining the impact of Chinese medicine regulation in the Australian state of Victoria supports the position that regulation enhances public safeguards with significant improvements in the management of consumer complaints and enforcement of professional standards after its inclusion in the statutory regulation scheme [106]. In addition, comparative examination of T&CM and non-T&CM professions in Australia’s National Registration and Accreditation Scheme has been shown to work at least as well as conventional medical practitioner regulation [107, 108].

Our review found that some T&CM practitioners perceived that professional associations adequately monitored and upheld professional standards obviating the need for regulation, a view contrary to stated consumer concerns. Examination of regulated and unregulated Australian T&CM professions indicates that self-regulatory mechanisms are not as effective for improving public protection when using T&CM services [109]. Furthermore, research on health care workforce regulatory frameworks and reforms emphasise the growing global focus on the public interest, a move away from self-regulatory governance models, and increased independence of complaints handling and oversight of disciplinary proceedings [27, 110].

As increasing T&CM use is a largely consumer-driven phenomenon (given T&CM is rarely integrated into public health systems), it is interesting that most consumers in this review stressed the need to regulate T&CM practices, a finding consistent with research conducted in 1996 (prior to the date inclusion of this review) [111]. This temporal consistency of consumer opinion suggests a long-standing public preference for the independent governance of health practitioners, including T&CM practitioners, who in this review were considered to require the same regulation as that governing conventional health practices. This is notable for two reasons. Firstly because consumer opinion regarding regulation persists despite the diversity of jurisdictions and T&CM professions examined, and secondly because these results run counter to research identifying temporal considerations of stakeholder analysis as potentially limited [112].

Although a common critique of T&CM regulation by some stakeholders is that regulation may grant these professions undue legitimacy, this view was not widely expressed in the identified studies. This finding, in addition to the worldwide reported rates of consumer use of [13–16], and trust in [113, 114] T&CM practices suggests that the issue of professional legitimacy may be immaterial to a significant proportion of consumers and health professionals who may already view these practices as legitimate regardless of regulatory status [115]. Additionally, the relevance of established accountabilities and minimum standards of a profession for reasons of public safety and debates about professional legitimacy should be viewed as separate and divorced issues.

While the findings suggest support for T&CM regulation across stakeholder groups, this may not necessarily indicate majority support. Support for or opposition to regulation of T&CM providers is highly contextual and based on factors such as integration, marginalisation, perceived professionalisation, competition, as well as the specific type of therapy or practice. For example, support for regulation from conventional health practitioners was clearly not universal. Physiotherapists and manual therapists in the Netherlands were less supportive of regulation of chiropractors. Conventional medicine professionals in Korea were also less supportive of legislative governance of acupuncture, chiropractic, and traditional Chinese medicine. These differences may be partly explained by perceived inter-professional competition, as indicated by policy-makers and conventional medicine associations, and implied by physiotherapists and manual therapists in this review. The above examples suggest a clear competitive tension in terms of similar scopes of practice. Such competitive tensions have also been suggested to exist between conventional medicine and naturopathy in Australian, Canadian, and German studies [116–118]. One commentator has proposed that the care offered by T&CM practitioners could be used to alleviate conventional care workforce shortages [119], a role which is broadly supported (where appropriate) by the WHO [9]. Competition between medical and non-medical clinicians has also been a long-standing issue with respect to policy development and integration beyond T&CM [120]. However, the degree to which T&CM-specific issues influence standard issues associated with inter-professional competitive tensions, and consequently issues such as views towards regulation and scope of practice of another health profession requires further research before it can be confirmed.

In this review lack of regulation was cited by numerous stakeholders as exposing patients to direct and indirect risks. Yet some GPs indicated that well-trained T&CM practitioners posed no risk of harm, and consumers indicated that T&CM treatments should only be prescribed by qualified providers. The issue of harm, therefore, may be a function of the competence of the practitioner and the quality of T&CM training [121, 122]. Supporting this argument is the uncertainty regarding T&CM practitioner competence and training standards which was frequently reported by stakeholders in this review, including T&CM practitioners. These, together with other non-health risks of T&CM practices, have been extensively reviewed by researchers with many of these risks purported to be exacerbated by lack of regulation [123]. These findings suggest that risks to consumers posed by T&CM practices may be mitigated by appropriate regulatory mechanisms that promote greater public protections via appropriate standards and accountability, while ensuring that consumer choice is protected, and practices can be appropriately integrated where there is evidence of patient benefit. In support of this, naturopathic education standards have been found to vary internationally, with nations that have workforce regulatory frameworks in place reporting higher and more consistent education standards than those without regulation [32].

As distinct from previously mentioned conflicting opinions between T&CM professions and consumers, there was also disagreement within the professions. The contrasting attitudes towards regulation between various T&CM practitioners, and between practitioners and organisational representatives may potentially be driven by professional self-interest. Several studies highlighted the benefits to and concerns of T&CM practitioners regarding regulation, revealing potential motivations of self-interest for attitudinal positions taken. ‘Self-interest’ may also be evident at the institutional level, particularly when organisations have been granted privileged roles within professions (such as through accredited or self-regulatory structures) that may be removed should those professions become statutorily regulated. This is suggested by previous examination, which has drawn attention to the resistance of much T&CM regulation in Australia being led by professional associations with commercial interests in educational institutions that may be adversely affected by the higher educational standards imposed by regulation [52, 124]. This view accords with researchers of T&CM and non-T&CM professions who contend that self-interest of education providers [125] and professional associations [126] are often incompatible with the public interest. As such, while T&CM stakeholder perspectives should be considered important context for development of policy, they should not be elevated above other stakeholder perspectives (particularly consumer expectations), and ultimately regulatory decisions should be guided by public interest arguments, and jurisdictional, health system and professional needs, irrespective of T&CM stakeholder views on regulation.

Despite a comprehensive search strategy, over two-thirds of included studies were conducted in three jurisdictions; Australia, Canada, and New Zealand. Yet these jurisdictions represent only a small part of global T&CM practice. High rates of consumer use of T&CM practices are reported in sub-Saharan Africa [17], South America [18], the Arab states [19], Asia [16], India [20], and other world regions [16] where traditional medicine is integral to many cultural health practices and beliefs. These regions are implementing widespread T&CM practitioner regulation [10] with little or no formal research examining stakeholder support, as evident from the review findings. Greater research focus is required into stakeholder opinions of regulation in these regions to ensure evidence-informed policy implementation efforts, and consistent application of regulatory measures across jurisdictions and practices.

Although most research on stakeholder attitudes and perceptions of T&CM regulation has been conducted in Australia, Canada, and New Zealand, this appears to have had limited impact as these jurisdictions have been amongst the most hesitant to implement consistent workforce regulation policies across T&CM professions [10, 31]. A striking example comes from Australia where herbal medicine and naturopathy has consistent stakeholder support for regulation, as well as having regulation recommended by every Commonwealth and State government review of the issue since 2000. Yet these professions remain self-regulated almost 50 years after the first government review recommending they be statutorily regulated [127]. Australia, like many countries, has placed much of its T&CM regulatory emphasis on product regulation, which the WHO considers is only part of the regulatory requirements it recommends to national governments [10]. This political inertia, described by some WHO member states as lack of ‘political will’ [10], also defies T&CM evidence of efficacy and demonstrable public health arguments for regulation of T&CM [9]. In addition to an increased global need for evidence to better inform regulatory decisions when implementing T&CM regulation, governments should also be accountable for ensuring that evidence, where it exists, is used to inform appropriate T&CM policy development. The most appropriate form of regulation for each health system, population, profession, and jurisdiction requires consideration based on evidence, and needs to be explored more explicitly. Our review indicates that regulation of TC&M is not being conducted in an evidence-informed manner. The juxtaposition of jurisdictions generating stakeholder research which has not translated into regulatory policy with jurisdictions implementing regulation without corresponding stakeholder evidence may have significant policy implications and requires careful consideration from the research community engaging with this topic.

## Limitations

This comprehensive review of the state of the current literature on the regulation of T&CM professions comes with several limitations which should be considered when interpreting the findings. Firstly, one-third of studies was identified through hand and grey literature searching. This was likely due to varying definitions, terms and practices used across jurisdictions making a systematic search more difficult. The lack of consensus on T&CM definitions resulted in considerable heterogeneity that was compounded by differences in jurisdictional definitions of conventional medicine versus T&CM practices, and inconsistencies regarding regulation across stakeholder groups. The use of collective T&CM terminology in some studies meant profession-specific data was not available. However, it is worth noting that T&CM is, by definition, a subjective term defined by the health system in which care is offered and as such this variability is understandable. Critical appraisal of qualitative studies was also problematic because there was no identified fit-for-purpose tool for the predominantly descriptive nature of these studies. The included papers cover an extensive time period and variation in attitudinal perspectives due to increasing use of T&CM practices may be expected over that time. However, temporal trends were generally not considered due to high heterogeneity across stakeholder groups as well as being considered of limited value in stakeholder analysis [112]. The results do not report on attitudes to the regulation of professions outside of T&CM such as conventional providers or hospital staff. Nor do the results report the impact of regulation on institutional stakeholders such as health insurers, accreditors, and product manufacturers, or on the health care system generally. Due to these factors, we can only make claims about attitudinal responses to specific professions for a select group of included studies during a specific time period, with a call for greater research into health care workforce regulation in order to explicate these factors. Notwithstanding these limitations, this work provides the most extensive review of this topic to date and may be useful for researchers and policy-makers seeking to examine or implement appropriate T&CM regulation in line with WHO recommendations.

## Conclusions

This systematic review identifies widespread consumer and practitioner support for the regulation of T&CM professions. Significantly, consumers and practitioners from all branches of health care are calling for greater independent governance of, and accountably for unregulated T&CM health care professions. The support for regulation derives from a need to safeguard the public by promoting practitioner competence through the establishment of professional and practice standards. Consumers, T&CM practitioners, and conventional medicine practitioners comprised the vast majority of identified studies and their opinions regarding regulation are well represented in the literature. However, there is little research on the views of professional associations, education providers and policy-makers, and no published research on institutional stakeholders such as health insurers and accreditors. The available empirical evidence suggests stakeholders largely support regulation, with policy-makers expecting certain professional commutations which may not be acceptable to T&CM practitioners. In order to corroborate the conclusions of this review, further research is required from a broader range of jurisdictions using rigorous research methods. Determining attitudes across the breadth of health care stakeholders is a critical first step in offering insights into the barriers and enablers of regulation, developing relevant policy and practice recommendations, and informing appropriate policy change regarding T&CM professional regulation.

## Data Availability

All data generated or analysed during this study are included in this published article and key sources are cited.

## Abbreviations

GPs: General practitioners
T&CM: Traditional and complementary medicine
WHO: World Health Organization
